# Tooth‐Specific Personalization of In‐Office Whitening Time: A Three‐Case Report

**DOI:** 10.1155/crid/9036041

**Published:** 2026-08-12

**Authors:** Matheus de Castro Costa, Priscila Toninatto Alves de Toledo, Diana Gabriela Soares

**Affiliations:** ^1^ Department of Operative Dentistry, Endodontics and Dental Materials, Bauru School of Dentistry, University of São Paulo (USP), São Paulo, Brazil, usp.br

**Keywords:** dentin, dentin hypersensitivity, enamel, tooth bleaching

## Abstract

Dental sensitivity during or after tooth whitening is a common adverse effect that may compromise patient comfort and treatment acceptance. This case report descriptively evaluated three patients who underwent in‐office whitening with a calcium‐containing 35% hydrogen peroxide gel, applied in three sessions at 7‐day intervals. A differential protocol was adopted, with reduced application time (from 40 to 20 min) according to tooth group, particularly for incisors, in an attempt to minimize sensitivity in teeth with reduced enamel and dentin thickness. Despite this adjustment, all patients reported some degree of sensitivity, predominantly in mandibular incisors, ranging from mild to severe. Notably, a homogeneous aesthetic outcome was achieved across different dental groups, even with reduced exposure times. Although color change and sensitivity distribution were assessed qualitatively, the findings provide clinical insight into the influence of anatomical variability on bleaching response. All patients reported high satisfaction with the aesthetic results. Within the limitations of this case report, reducing application time alone was insufficient to control sensitivity, suggesting that additional strategies may be required, particularly for thinner teeth. This approach may support the development of more individualized bleaching protocols based on dental characteristics.

## 1. Introduction

Dental aesthetics are closely associated with quality of life and self‐esteem, as they directly influence self‐confidence and social well‐being. In this context, tooth whitening has become a widely adopted, minimally invasive approach to enhance smile appearance, using agents such as carbamide peroxide and hydrogen peroxide (HP), available in different concentrations for both at‐home and in‐office protocols [1, 2]. Despite its effectiveness, tooth sensitivity remains the most frequently reported adverse effect of bleaching procedures. Pain intensity is influenced by multiple factors, including the concentration of the bleaching agent, its contact time with dental tissues, and the pH of the formulation [3]. HP can rapidly diffuse through enamel and dentin, reaching the pulp chamber and triggering inflammatory responses associated with pain [4, 5]. This diffusion has been confirmed by both color changes in cross‐sectional specimens and detection of HP within the pulp chamber [6]. Histopathological studies further demonstrate superficial pulp necrosis in teeth with reduced enamel and dentin thickness, such as mandibular incisors, following the application of high‐concentration HP [7–9].

Enamel and dentin thickness plays a critical role in both bleaching efficacy and dental sensitivity. Reduced thickness is associated with increased sensitivity but also with faster whitening, likely due to enhanced diffusion of reactive species through a thinner mineralized barrier [10–12]. Based on these considerations, bleaching protocols should be individualized according to anatomical characteristics, including enamel/dentin thickness, pulp chamber volume, patient age, and baseline discoloration [13]. Accordingly, sensitivity intensity should guide modifications in bleaching protocols. Increasing attention has been directed toward strategies that reduce treatment time and exposure to whitening agents while maintaining clinical efficacy [3]. In this context, calcium‐containing high‐concentration in‐office bleaching agents, characterized by a more alkaline pH, have been associated with lower incidence and intensity of sensitivity [14, 15]. However, clinical evidence remains limited regarding whether adjusting bleaching exposure time across different dental groups within the same patient can influence sensitivity patterns while preserving aesthetic outcomes. Therefore, this case report is aimed at evaluating tooth sensitivity as the primary outcome in response to reduced bleaching exposure times across different dental groups within the same patient and secondarily assessing color change and patient‐reported aesthetic perception.

## 2. Case Report

The following three clinical cases were conducted following the CARE case report guidelines and using a standardized bleaching protocol, with tooth color assessment and sensitivity evaluation performed consistently across all patients. Patients with age from 20 to 30 years and with (i) no gingival resection, (ii) no active caries, (iii) no erosion, (iv) no restorations on the teeth surface selected for bleaching, (v) no pre‐existing dentin hypersensitivity, (vi) no use of desensitizing agents prior to treatment, and (vii) no systemic or behavioral factors that could influence pain perception were selected. Written informed consent for publication of the case details and any accompanying images was obtained from all patients included in this report.

### 2.1. Initial Color Assessment

Professional prophylaxis was performed on all teeth scheduled for whitening using a Robson brush with pumice and water. Following rinsing and gentle air‐drying, while maintaining slight surface moisture, baseline tooth color was recorded using a standardized VITA Classical shade guide, as commonly applied in bleaching studies. The shade guide tabs were arranged according to value (from highest to lowest brightness: B1, A1, B2, D2, A2, C1, C2, D4, A3, D3, B3, A3.5, B4, C3, A4, and C4), and color matching was performed under standardized lighting conditions by visual comparison of the middle third of the buccal surface. Color assessments were repeated 1 week after each whitening session to monitor treatment effectiveness over time. The same operator performed all color assessments.

### 2.2. Whitening Protocol

**Figure 1:**
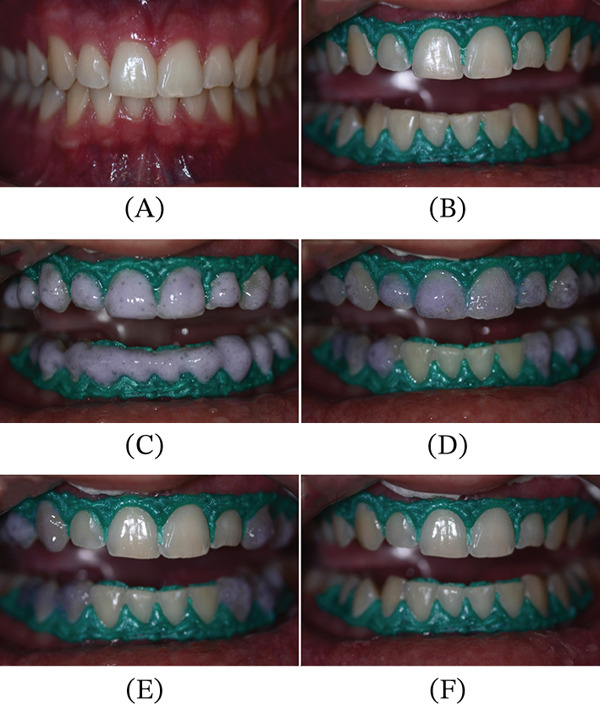
Dental whitening protocol: illustrative sequence of the main procedural steps. (A) Initial clinical assessment; (B) gingival isolation with light‐cured resin for soft tissue protection; (C) application of the whitening agent to the tooth surfaces; (D) removal of the whitening agent from the lower incisors after 20 min; (E) removal of the whitening agent from the upper lateral incisors after 25 min and from the upper central incisors after 30 min; (F) removal of the whitening agent from the upper and lower canines, as well as upper and lower premolars, after 40 min.

The whitening procedure was performed from the second premolar to the contralateral second premolar (Figure 1). No desensitizing agents were used during the bleaching procedures in order to avoid potential interference with treatment efficacy and to allow a direct assessment of tooth sensitivity associated with different bleaching exposure times. This approach was adopted to minimize confounding factors and to evaluate whether the proposed protocol could reduce sensitivity without the need for additional clinical steps. Prior to gel application, relative isolation was achieved with cotton rolls and continuous suction. The gingival tissues were carefully dried, and a light‐cured resin barrier (Top Dam, FGM, Joinville, Brazil) was applied along the cervical margins of the teeth. The barrier extended approximately 3–5 mm onto the attached gingiva and interproximal papillae, and 1 mm onto the cervical portion of the teeth, ensuring complete sealing of the marginal gingiva. The barrier was applied in a continuous layer with sufficient thickness to prevent leakage of the bleaching gel and to provide mechanical protection to the soft tissues. Each segment was polymerized for 20 s using a LED curing unit (DB 685, Dabi Atlante, 1100 mW/cm^2^), and the integrity and adaptation of the barrier were verified with an exploratory probe prior to initiating the bleaching procedure to ensure adequate isolation and patient safety.

The treatment protocol consisted of three in‐office sessions performed at 7‐day intervals, allowing assessment of color change and reducing the risk of discomfort associated with tooth sensitivity. A 35% HP gel containing calcium (Whiteness HP Blue, FGM, Joinville, SC, Brazil) was used according to the manufacturer′s instructions. The product was supplied in a two‐component system (HP and thickener) with a syringe coupling system, which allowed the components to be mixed immediately prior to application to obtain a homogeneous gel with uniform consistency and coloration, ensuring standardized concentration and handling characteristics.

After manipulation, the gel was applied in a uniform layer approximately 1 mm thick over the buccal surfaces of the teeth using a syringe tip, covering the vestibular surface and avoiding excess near the gingival barrier. Care was taken to standardize the amount of gel and ensure complete coverage of the enamel surface within each tooth group. In each session, a single application was performed, with total exposure time adjusted according to the estimated enamel and dentin thickness of each group: mandibular incisors (20 min; Figure 1D), maxillary lateral incisors (25 min; Figure 1E), maxillary central incisors (30 min; Figure 1E), and maxillary and mandibular canines and premolars (40 min; Figure 1F). The bleaching gel was selectively removed from each dental group according to the predetermined exposure time, without affecting adjacent groups. Initially, the excess gel was aspirated using an endodontic suction tip. Residual material was then carefully removed with a cotton pellet moistened with water, followed by a dry cotton pellet. This procedure was performed locally for each dental group, ensuring that adjacent areas remained unaffected.

At the end of each bleaching session, the whitening gel was carefully removed using high‐volume suction, followed by thorough rinsing with an air–water spray to eliminate any residual material from the tooth surfaces and gingival barrier. The gingival barrier was then gently detached using an exploratory instrument, and the integrity of the soft tissues was clinically inspected. Subsequently, the enamel surfaces were polished using a fine polishing paste and a soft rubber cup at low speed to remove superficial residues and enhance surface smoothness. This step was performed under light pressure and intermittent motion to avoid heat generation. After polishing, the teeth were rinsed and gently air‐dried, and the patient received postoperative instructions regarding dietary precautions and the management of possible sensitivity.

### 2.3. Sensitivity Assessment

Tooth sensitivity related to the whitening procedure was monitored using the Numerical Rating Scale (NRS) (de Paula et al. [16]). The NRS categorizes sensitivity as follows: 0: no sensitivity, 1: mild sensitivity, 2: moderate sensitivity, 3: considerable sensitivity, and 4: severe sensitivity. Patients were requested to report the level of sensitivity during the procedure, immediately after each session, and within 48 h after treatment. This assessment was conducted at every whitening session to monitor sensitivity progression throughout the treatment.

### 2.4. Treatment Satisfaction Assessment

Patient‐reported satisfaction with the aesthetic outcomes of the tooth whitening treatment was evaluated at the end of each session using a standardized subjective assessment approach commonly applied in clinical studies. Participants were asked to rate their overall satisfaction with the treatment results. Responses were recorded immediately after each session to capture real‐time perceptions and to monitor changes in satisfaction throughout the treatment period.

## 3. Case 1: Clinical Report

A 20‐year‐old male patient presented to the dental clinic at the Bauru School of Dentistry, University of São Paulo, reporting dissatisfaction with the color of his teeth. Medical history revealed no systemic conditions and no habits associated with extrinsic staining, such as frequent coffee consumption or smoking. The patient reported no previous history of tooth whitening treatment nor baseline sensitivity.

**Figure 2:**
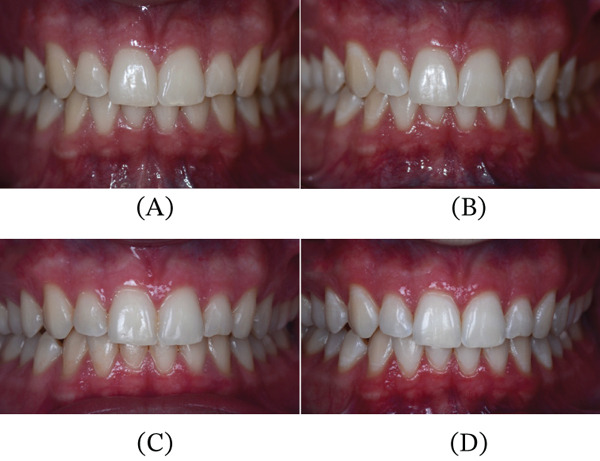
Clinical progression of tooth whitening on Case 1. (A) Initial clinical appearance; (B) after the first in‐office whitening session; (C) after the second session; (D) final result following the third session.

Initial color assessment, performed using the VITA classical shade guide, revealed shade A1 for the maxillary central incisors and shade A2 for the mandibular incisors, maxillary lateral incisors, canines, and premolars. The whitening procedure was carried out according to the previously described protocol (Figure 1). After the first session, the patient reported mild tooth sensitivity (Score 1) in teeth 31 and 32 during gel application. A slight improvement in tooth color was observed; however, the desired aesthetic outcome had not yet been achieved. During the second session, mild sensitivity (Score 1) was again reported, affecting teeth 32 and 12. In the third session, the patient reported moderate sensitivity (Score 2), involving teeth 21, 31, 41, and 42. Final color achieved was A1 for mandibular and maxillary incisors and premolars, and A2 for canines. The patient expressed high satisfaction with the aesthetic outcomes obtained across all tooth groups (Figure 2). Nevertheless, satisfaction regarding the level of sensitivity experienced during the procedure was rated as moderate.

## 4. Case 2: Clinical Report

**Figure 3:**
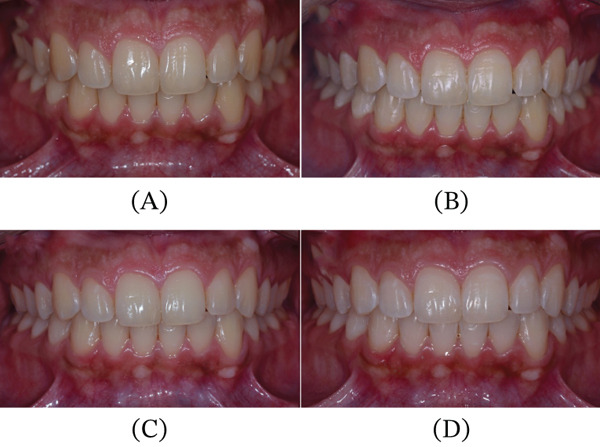
Clinical progression of tooth whitening on Case 2. (A) Initial clinical appearance; (B) after the first in‐office whitening session; (C) after the second session; (D) final result following the third session.

A 23‐year‐old female patient presented to the dental clinic at Bauru School of Dentistry, University of São Paulo, complaining of yellowish tooth discoloration, which she attributed to frequent coffee consumption. The anamnesis revealed no relevant systemic conditions, and the patient denied smoking habits. After professional prophylaxis and comprehensive intraoral examination, shade assessment using the VITA classical shade guide revealed shade A3 for the maxillary and mandibular incisors and shade A3.5 for the maxillary and mandibular canines and premolars (Figure 3). The patient reported no tooth sensitivity at baseline.

Following the first whitening session, the patient reported sensitivity (Score 2) in tooth 41 during the first hours after treatment. No sensitivity was reported during the second session (Score 0). However, sensitivity in tooth 41 recurred during the third session, with considerable intensity (Score 3). All incisors (mandibular and maxillary) reached A1 color at the end of treatment, whereas premolars and canines reached B2. Despite the sensitivity episodes, the patient expressed high satisfaction with the final aesthetic outcome (Figure 3), although satisfaction regarding the sensitivity experienced during and after the procedure was rated as moderate.

## 5. Case 3: Clinical Report

A 30‐year‐old male patient presented to the dental clinic at Bauru School of Dentistry, University of São Paulo, reporting dissatisfaction with the color of his teeth and requesting whitening treatment. His medical history revealed no relevant systemic conditions, and he denied coffee consumption and smoking. Supragingival calculus was observed in the interproximal areas of the mandibular incisors, particularly on the lingual surfaces, although periodontal tissues were generally healthy. The patient reported no tooth sensitivity prior to treatment.

**Figure 4:**
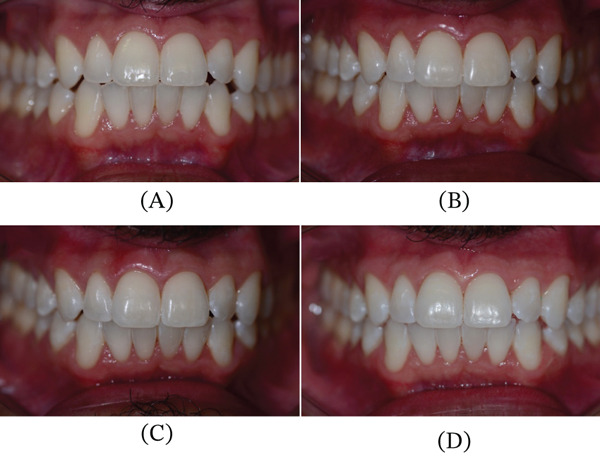
Clinical progression of tooth whitening on Case 3. (A) Initial clinical appearance; (B) after the first in‐office whitening session; (C) after the second session; (D) final result following the third session.

Initial procedures included scaling and professional prophylaxis. Baseline shade assessment, performed using the VITA classical shade guide, revealed shade A1 for the maxillary and mandibular incisors and shade A2 for the remaining tooth groups. The same bleaching material and protocol previously described were applied in this case. After the first session, the patient reported severe sensitivity (Score 4) in the mandibular incisors (31, 32, 41, and 42), describing the sensation as painful. Three hours after the session, sensitivity decreased to Score 1, and after 24 h, no sensitivity was reported (Score 0). During the second session, the patient reported mild sensitivity on mandibular incisors region (Score 1) but was unable to identify a specific tooth. No sensitivity was reported between 3 and 24 h post‐treatment. At the end of treatment, the final color was B1 for incisors and A1 for canine and premolars. Despite the sensitivity episodes, the patient expressed high satisfaction with the final shade achieved (Figure 4) and moderate satisfaction regarding the sensitivity experienced during treatment.

## 6. Discussion

In‐office treatments with high‐concentration HP produce faster visible results, whereas at‐home protocols require longer application periods. Nevertheless, both approaches tend to result in similar final color changes [17]. In the present case report, adjusting bleaching exposure time according to tooth group allowed controlled application while maintaining clinical effectiveness. A progressive improvement in tooth color was observed after each session, and importantly, a homogeneous aesthetic outcome was achieved across different dental groups, despite reduced exposure times in thinner teeth. However, tooth sensitivity was still reported in all patients, predominantly affecting mandibular incisors. These findings suggest that, although exposure time modulation may contribute to aesthetic standardization, it alone is insufficient to prevent sensitivity.

The bleaching agent used contained 2% calcium gluconate, which has been associated with reduced enamel demineralization and lower postoperative sensitivity. This effect may be attributed to the formation of calcium hydroxide and a more stable alkaline pH, which can limit peroxide diffusion and dentin permeability [6, 15]. Clinical evidence supports this, showing similar bleaching efficacy but significantly lower sensitivity in calcium‐containing formulations compared with calcium‐free gels [14]. However, in the present cases, all patients still reported some degree of sensitivity, albeit generally mild and transient. The mechanisms underlying bleaching‐related sensitivity are multifactorial and may involve oxidative damage and pulp inflammation, hydrodynamic fluid movement within dentinal tubules due to oxygen bubble formation, and direct nociceptor activation by HP [3, 18]. In addition, the persistence of sensitivity may be explained by the rapid diffusion of HP, particularly in thinner teeth, as well as individual variability in pulpal inflammatory response and neural sensitivity thresholds [4, 5]. Together, these factors help explain why reducing exposure time alone was insufficient to eliminate sensitivity, especially in mandibular incisors.

Case 1 exhibited a greater number of sensitive teeth, with low scores, whereas in the other cases, higher intensity was reported in isolated mandibular incisors. No participant reported prolonged sensitivity beyond the initial postoperative period, although one instance of severe sensitivity occurred during the first session. These findings are consistent with previous reports showing that even highly concentrated HP gels can induce mild to intense sensitivity within the first 12 h after treatment, especially in mandibular incisors [19]. Importantly, the patients in this case report were young adults, a group characterized by wider dentinal tubules, thinner dentin, and reduced tertiary dentin deposition [8]. Enamel and dentin thickness plays a critical role in both bleaching efficacy and dental sensitivity, as reduced thickness is associated with increased sensitivity but also with faster whitening effectiveness [10–12]. This pattern was also observed in the present study, in which patients aged 20–30 years achieved satisfactory color outcomes despite reduced bleaching time, while still reporting sensitivity, particularly in mandibular incisors, which present lower enamel and dentin thickness. These anatomical features favor faster diffusion of reactive oxygen species through enamel and dentin, increasing the likelihood of pulpal inflammatory response [12]. Although modern formulations include desensitizing and buffering agents, these components do not completely eliminate the oxidative and cytotoxic effects of HP [20].

Computed tomography analysis performed by Esteves et al. [12] demonstrated significant anatomical differences among tooth groups, with premolars exhibiting the greatest hard tissue thickness and pulp volume, followed by canines, maxillary incisors, and mandibular incisors. These structural variations directly influence HP behavior during bleaching. Mandibular incisors showed six‐fold greater peroxide diffusion compared with premolars, whereas premolars presented a pulp volume nine times larger than mandibular incisors. Additionally, incisors achieved color saturation one session earlier than canines and premolars. Collectively, these findings indicate that HP diffusion is inversely proportional to dental thickness and that anatomical characteristics significantly affect both bleaching kinetics and whitening outcomes, with thinner teeth demonstrating faster diffusion and earlier color stabilization. Another study demonstrated that central and lateral incisors exhibit reduced buccal enamel–dentin thickness compared with canines, which was associated with increased HP penetration into the pulp chamber following in‐office bleaching [21]. This pattern is consistent with the present findings, in which mandibular incisors showed the highest recurrence of sensitivity, followed by premolars. Notably, in one case where sensitivity was reported in a premolar, the presence of an enamel crack was observed, which may have contributed to increased peroxide penetration and heightened sensitivity.

The biological effects on the dental pulp, clinically manifested as tooth sensitivity, appear to follow the same anatomical trend. Thinner enamel and dentin facilitate the penetration of reactive oxygen species, increasing oxidative stress within pulpal tissue. In vitro studies support this mechanism, demonstrating pulpal cell damage after exposure to bleaching agents, with enamel/dentin thickness and gel contact time playing critical roles [10]. Histopathological analyses of human teeth subjected to conventional in‐office bleaching protocols further corroborate these findings: Premolars generally show no significant pulpal alterations or sensitivity [22], whereas mandibular incisors exhibit superficial necrotic areas, inflammatory infiltrate, and tertiary dentin deposition as a reparative response to injury [7–9]. Together, this evidence reinforces the influence of anatomical characteristics on pulpal response and underscores the importance of limiting gel contact time to minimize adverse effects [20].

Although the findings demonstrated consistent color outcomes, some limitations should be considered. The use of a visual shade guide instead of a spectrophotometer may have reduced the objectivity of color assessment; however, this approach reflects routine clinical practice, and standardized lighting conditions and examiner calibration were adopted to minimize subjectivity. Additionally, photographs were obtained immediately after bleaching, without follow‐up assessments. This may have led to a slight overestimation of whitening due to transient enamel dehydration caused by HP. The absence of longer term photographic evaluation was mainly due to patient compliance limitations, which restricted return visits for follow‐up. Nevertheless, color was considered a secondary outcome, whereas the primary focus of the study was tooth sensitivity.

Based on the present findings and available evidence, bleaching protocols should be individualized according to dental anatomy. Reducing application time for thinner teeth allowed achievement of homogeneous whitening while helping control sensitivity. Nevertheless, a 20‐min application was insufficient to eliminate sensitivity in mandibular incisors, which was reported as moderate to severe in two cases, suggesting the need for a less aggressive protocol for this tooth group. Novel protocols using low‐concentration HP bleaching gels have emerged as alternatives for tooth whitening, and their combination with violet LED irradiation has been proposed to enhance efficacy while minimizing cytotoxicity and patient‐reported sensitivity [20, 23]. Additionally, the adjunctive use of desensitizing agents, such as potassium nitrate combined with sodium fluoride or arginine, may further reduce discomfort without compromising whitening outcomes [24]. Overall, the results highlight that bleaching efficacy and sensitivity are influenced not only by gel concentration and composition but also by anatomical characteristics of each tooth group, reinforcing the need for tailored clinical protocols.

## 7. Conclusion

Based on the present findings, modification of the bleaching protocol resulted in a partial reduction in tooth sensitivity in some dental groups, although discomfort persisted, particularly in mandibular incisors. From an aesthetic perspective, reducing application time did not compromise the final whitening outcome or patient satisfaction. These findings should be interpreted with caution, as they are based on a limited number of cases and do not allow for definitive clinical recommendations. Rather than establishing a clinical alternative, this approach may be considered exploratory and hypothesis generating, providing preliminary insights into the relationship between bleaching time, dental anatomy, and sensitivity. Further well‐designed controlled clinical studies are required to validate these observations and determine their clinical relevance.

## Author Contributions

Matheus de Castro Costa: conceptualization, investigation, data curation, clinical procedures, writing – original draft preparation. Priscila Toninatto Alves de Toledo: investigation, data curation, writing – review and editing. Diana Gabriela Soares: conceptualization, methodology, supervision, validation, writing – review and editing, project administration.

## Funding

This work is supported by the Coordination for the Improvement of Higher Education Personnel (CAPES) under Finance Code 001.

## Disclosure

All authors reviewed and approved the final version of the manuscript and agree to be accountable for all aspects of the work, ensuring that questions related to the accuracy or integrity of any part of the work are appropriately investigated and resolved.

## Conflicts of Interest

The authors declare no conflicts of interest.

## Data Availability

The data that support the findings of this study are available from the corresponding author upon reasonable request.
